# Supplementary material for: Whole genomic analysis reveals atypical non-homologous off-target large structural variants induced by CRISPR-Cas9-mediated genome editing
Source: Nat Commun. 2023 Aug 25;14:5183. doi: 10.1038/s41467-023-40901-x (PMC10457329; doi:10.1038/s41467-023-40901-x)
Supplement: Supplementary file 1 — Supplementary Information [file 41467_2023_40901_MOESM1_ESM.pdf]

Whole genomic analysis reveals atypical non-homologous off-target large structural variants induced by CRISPR-Cas9-mediated genome editing

Supplementary information

Supplementary Table 1.

Sequencing data statistics of parental and edited genomes.

Lines	DNA in molecule >20 Kb	Mean depth	Mapping rate	% SNPs phased	Number of reads
NC01	90.8%	56.2x	95.5%	99.2%	1,318,618,290
NC01-*B2M*$^{-/-}$-1	93.9%	53.4x	95.4%	99.1%	1,255,152,084
NC01-*B2M*$^{-/-}$-2	95.3%	48.5x	95.5%	99.1%	1,137,678,996
NC01-*B2M*$^{-/-}$-3	90.3%	53.1x	95.3%	99.1%	1,252,163,658

Average mean depth: 52.8x; Average mapping rate: 95.4%; Average % SNPs phased: 99.1%

Supplementary Table 1. Sequencing data statistics of parental and edited genomes. The HMW gDNAs of parental and edited clones were extracted and subjected to 10x Genomics Linked-Reads Chromium whole genome sequencing and aligned with GRCh38.

Supplementary Table 2.

List of potential off-target sites predicted by Cas-OFFinder with mismatch numbers equal to or less than 6 and DNA bulge size equal to or less than 2, which are close to the large SV1 (chr3:41537429-41673419) detected in the *B2M* knockout clone*.

Chromosome	Genome localization	Strand	Mismatches	Bulge Size
chr3	40913774	+	5	0
chr3	45606634	-	5	0

*These predicted target sites are too far (0.6- and 3.9 Mb, respectively) away from the large SV to account for Cas9 cleavage.

Supplementary Table 3.

List of potential off-target sites predicted by CRISTA, which are close to the large SV (chr3:41537429-41673419) detected in the *B2M* knockout clone*.

chromosome	strand	start position	pairwise alignment score	total_bulges	#mismatches	CRISTA score
chr3	+	40010226	13.5	2	2	0.145146828
chr3	+	46541862	12.5	2	3	0.174546442

*These predicted target sites are too far (0.6- and 4.7Mb, respectively) away from the large SV to account for Cas9 cleavage.

Supplementary Table 4.

List of potential off-target sites predicted by Elevation, which are close to the large SV (chr3:41537429-41673419) detected in the *B2M* knockout clone*.

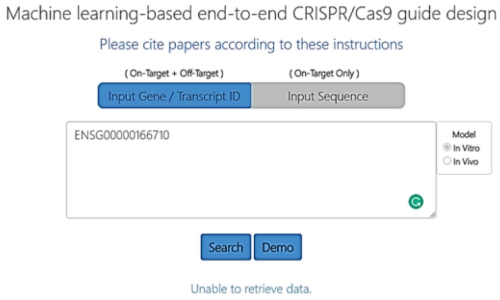

* Elevation software was interrogated for *B2M* gene (ENSG00000166710), it showed “unable to retrieve data”.

Supplementary Table 5.

Sequencing data statistics of parental and edited genomes.

Lines	DNA in molecule >20 Kb	DNA in molecule >100 Kb	Mean depth	Mapping rate	% SNPs phased	Number of reads
iPSC-71	95.5%	20.4%	57.4x	97.7%	99%	1,294,721,622
iPSC-71-$APP^{C/G}$	97.3%	70.5%	56.3x	97%	99%	1,277,632,416
iPSC-71-$APP^{C/C}$	97%	71.7%	60.4x	96.9%	99%	1,372,945,614

Average mean depth: 58x; Average mapping rate: 97.2%; Average % SNPs phased: 99%

Supplementary Table 5. Sequencing data statistics of parental and edited genomes. The HMW gDNAs of parental and edited clones were extracted and subjected to 10x Genomics Linked-Reads Chromium whole genome sequencing and aligned with GRCh38.

Supplementary Table 6.

List of potential off-target sites predicted by Cas-OFFinder with mismatch numbers equal to or less than 6 and DNA bulge size equal to or less than 2, which are close to the region of large SV (chr3:39882164-39973392) detected in the *APP*$^{C/C}$ clone*.

Chromosome	Genome localization	Strand	Mismatches	Bulge Size
chr3	39771442	+	3	0
chr3	39468214	-	4	0
chr3	40570665	-	4	0

*These predicted target sites are too far (0.1-, 0.4-, and 0.59 Mb, respectively) away from the large SV to account for Cas9 cleavage.

Supplementary Table 7.

List of potential off-target sites predicted by CRISTA, which are close to the large SV (chr3:39882164-39973392) detected in the *APP*$^{C/C}$ clone*.

chromosome	strand	start position	pairwise alignment score	total_bulges	#mismatches	CRISTA score
chr3	+	39416247	12.25	3	1	0.223678018
chr3	-	39468215	16	0	4	0.266102458
chr3	+	39771442	14.75	1	3	0.098162792
chr3	-	40570666	14.75	1	3	0.083083998

*These predicted target sites are too far (0.46-, 0.4-, 0.1- and 0.59 Mb, respectively) away from the large SV to account for Cas9 cleavage.

Supplementary Table 8.

List of potential off-target sites predicted by Elevation, which are close to the large SV (chr3:39882164-39973392) detected in the *APP*$^{C/C}$ clone*.

Chromosome	Position	Offtarget	Gene	Guide-Off-Target Score
chr3	39771441	AGAAAATTCTGCCATGACTCAGG	NoGene	0.8604388
chr3	40570666	ACCAAATTCCAACCTGACTCTGG	ZNF621	0.9256123

*These predicted target sites are too far (0.1- and 0.59 Mb, respectively) from the large SV to account for Cas9 cleavage.

Supplementary Table 9.

Sequencing data statistics of parental and edited genomes.

Lines	DNA in molecule >20 Kb	DNA in molecule >100 Kb	Mean depth	Mapping rate	% SNPs phased	Number of reads
H9	96.9%	67.2%	55.1x	96.4%	99.5%	1,256,467,662
H9-*B3GALT5*$^{-/-}$-1	96.2%	62.9%	45.2x	95.6%	99.4%	1,046,826,634
H9-*B3GALT5*$^{+/-}$-1	96.2%	67.7%	55.7x	95.3%	99.4%	1,294,977,400
H9-*B3GALT5*$^{-/-}$-2	97.3%	68.1%	51.2x	97.7%	99.3%	1,177,286,556
H9-*B3GALT5*$^{-/-}$-3	97.3%	70.6%	50.7x	96.3%	99.3%	1,171,568,656
ND40019*C	89.5%	38.4%	47.1x	94.9%	98.6%	1,116,487,912
LRRK2 (G2019S)-7	85.2%	14%	49.9x	95.4%	98.7%	1,181,333,672
LRRK2 (G2019S)-35	86%	20.9%	48.4x	96.3%	98.8%	1,120,213,700
iPSC-71-*DSG2*-1	96.2%	62.8%	58.3x	94.9%	99.4%	1,380,065,018
iPSC-71-*DSG2*-2	95.6%	61.2%	59.0x	94.3%	99.4%	1,412,439,810

Supplementary Table 10. List of potential off-target sites predicted by CasOFFinder, which are too far away from the chr2p16-22 translocation (chr2:36300001-60548383) to account for Cas9-DNA cleavage.

sgRNA	Chromosome	Position	Direction	Mismatches	Bulge Size
CACACAGATCTACAAGGCCC (B0702-ex2g1)	chr2	35257066	-	2	1
CACACAGATCTACAAGGCCC (B0702-ex2g1)	chr2	35257066	-	3	1
CACACAGATCTACAAGGCCC (B0702-ex2g1)	chr2	38837708	-	3	1
CACACAGATCTACAAGGCCC (B0702-ex2g1)	chr2	38837708	-	3	1
CACACAGATCTACAAGGCCC (B0702-ex2g1)	chr2	42282945	-	3	1
CACACAGATCTACAAGGCCC (B0702-ex2g1)	chr2	43066460	+	3	1
CACACAGATCTACAAGGCCC (B0702-ex2g1)	chr2	43066460	+	3	1
CACACAGATCTACAAGGCCC (B0702-ex2g1)	chr2	44948237	-	3	2
CACACAGATCTACAAGGCCC (B0702-ex2g1)	chr2	45150133	+	3	1
CACACAGATCTACAAGGCCC (B0702-ex2g1)	chr2	45793602	-	4	0
CACACAGATCTACAAGGCCC (B0702-ex2g1)	chr2	47537943	+	3	1
CACACAGATCTACAAGGCCC (B0702-ex2g1)	chr2	47551897	-	3	1
CACACAGATCTACAAGGCCC (B0702-ex2g1)	chr2	51092483	-	3	1
CACACAGATCTACAAGGCCC (B0702-ex2g1)	chr2	51616623	+	3	1
CACACAGATCTACAAGGCCC (B0702-ex2g1)	chr2	51616623	+	3	1
CACACAGATCTACAAGGCCC (B0702-ex2g1)	chr2	54601355	-	3	1
CACACAGATCTACAAGGCCC (B0702-ex2g1)	chr2	56038698	-	4	0
CACACAGATCTACAAGGCCC (B0702-ex2g1)	chr2	56038698	-	3	1
CACACAGATCTACAAGGCCC (B0702-ex2g1)	chr2	56038698	-	3	1
CACACAGATCTACAAGGCCC (B0702-ex2g1)	chr2	56038698	-	2	2
CACACAGATCTACAAGGCCC (B0702-ex2g1)	chr2	56038698	-	2	2
CACACAGATCTACAAGGCCC (B0702-ex2g1)	chr2	56038698	-	2	2
CACACAGATCTACAAGGCCC (B0702-ex2g1)	chr2	56038698	-	2	2
CACACAGATCTACAAGGCCC (B0702-ex2g1)	chr2	56038698	-	2	2
CACACAGATCTACAAGGCCC (B0702-ex2g1)	chr2	57900070	+	3	1
CACACAGATCTACAAGGCCC (B0702-ex2g1)	chr2	57900070	+	3	1
CACACAGATCTACAAGGCCC (B0702-ex2g1)	chr2	57900070	+	3	1
CACACAGATCTACAAGGCCC (B0702-ex2g1)	chr2	57925211	-	2	2
CACACAGATCTACAAGGCCC (B0702-ex2g1)	chr2	60634249	+	3	2
GATCTGAGCCGCGGTGTCCG (C0702-ex3g3)	chr2	33976808	+	4	1
GATCTGAGCCGCGGTGTCCG (C0702-ex3g3)	chr2	33976808	+	4	1
GATCTGAGCCGCGGTGTCCG (C0702-ex3g3)	chr2	35112139	+	5	0
GATCTGAGCCGCGGTGTCCG (C0702-ex3g3)	chr2	37272893	+	5	0
GATCTGAGCCGCGGTGTCCG (C0702-ex3g3)	chr2	37978073	-	4	2
GATCTGAGCCGCGGTGTCCG (C0702-ex3g3)	chr2	39628942	-	4	2
GATCTGAGCCGCGGTGTCCG (C0702-ex3g3)	chr2	42359127	-	3	2
GATCTGAGCCGCGGTGTCCG (C0702-ex3g3)	chr2	42394303	-	4	2
GATCTGAGCCGCGGTGTCCG (C0702-ex3g3)	chr2	42394303	-	3	2
GATCTGAGCCGCGGTGTCCG (C0702-ex3g3)	chr2	42622157	-	4	2
GATCTGAGCCGCGGTGTCCG (C0702-ex3g3)	chr2	42896089	+	4	2
GATCTGAGCCGCGGTGTCCG (C0702-ex3g3)	chr2	43063760	-	4	2
GATCTGAGCCGCGGTGTCCG (C0702-ex3g3)	chr2	43678462	-	4	2
GATCTGAGCCGCGGTGTCCG (C0702-ex3g3)	chr2	43678462	-	4	2
GATCTGAGCCGCGGTGTCCG (C0702-ex3g3)	chr2	44068086	-	4	2
GATCTGAGCCGCGGTGTCCG (C0702-ex3g3)	chr2	45030064	-	5	0
GATCTGAGCCGCGGTGTCCG (C0702-ex3g3)	chr2	45030064	-	3	1
GATCTGAGCCGCGGTGTCCG (C0702-ex3g3)	chr2	45713251	-	3	2
GATCTGAGCCGCGGTGTCCG (C0702-ex3g3)	chr2	46792947	-	3	2
GATCTGAGCCGCGGTGTCCG (C0702-ex3g3)	chr2	46792947	-	3	2
GATCTGAGCCGCGGTGTCCG (C0702-ex3g3)	chr2	46912055	-	3	2
GATCTGAGCCGCGGTGTCCG (C0702-ex3g3)	chr2	49742943	+	5	0
GATCTGAGCCGCGGTGTCCG (C0702-ex3g3)	chr2	54368735	-	5	0
GATCTGAGCCGCGGTGTCCG (C0702-ex3g3)	chr2	54485430	+	4	2
GATCTGAGCCGCGGTGTCCG (C0702-ex3g3)	chr2	56106176	+	5	0
GATCTGAGCCGCGGTGTCCG (C0702-ex3g3)	chr2	56106176	+	3	2
GATCTGAGCCGCGGTGTCCG (C0702-ex3g3)	chr2	56556885	+	5	0
GATCTGAGCCGCGGTGTCCG (C0702-ex3g3)	chr2	56556887	+	3	2
GATCTGAGCCGCGGTGTCCG (C0702-ex3g3)	chr2	60149897	-	5	0
GATCTGAGCCGCGGTGTCCG (C0702-ex3g3)	chr2	60334152	-	4	1
GATCTGAGCCGCGGTGTCCG (C0702-ex3g3)	chr2	60385253	+	5	0
GATCTGAGCCGCGGTGTCCG (C0702-ex3g3)	chr2	61258977	-	5	0

Supplementary Table 11.

List of potential off-target sites predicted by CRISTA, which are too far away from the chr2p16-22 translocation (chr2:36300001-60548383) to account for Cas9-DNA cleavage.

sgRNA	chromosome	strand	start position	pairwise alignment score	total_bulges	#RNA bulges	#DNA bulges	#mismatches	CRISTA score
CACACAGATCTACAAGGCCC (B0702-ex2g1)	chr2	-	38837709	14.75	1	1	0	3	0.129116
CACACAGATCTACAAGGCCC (B0702-ex2g1)	chr2	-	42282946	14.75	1	1	0	3	0.110502
CACACAGATCTACAAGGCCC (B0702-ex2g1)	chr2	+	43066461	14.75	1	1	0	3	0.087689
CACACAGATCTACAAGGCCC (B0702-ex2g1)	chr2	+	45150134	14.75	1	1	0	3	0.098652
CACACAGATCTACAAGGCCC (B0702-ex2g1)	chr2	+	45611338	12.5	2	2	0	3	0.130078
CACACAGATCTACAAGGCCC (B0702-ex2g1)	chr2	+	47537944	14.75	1	1	0	3	0.086638
CACACAGATCTACAAGGCCC (B0702-ex2g1)	chr2	-	47551898	15.75	1	0	1	3	0.169851
CACACAGATCTACAAGGCCC (B0702-ex2g1)	chr2	-	56038699	16	0	0	0	4	0.33646
CACACAGATCTACAAGGCCC (B0702-ex2g1)	chr2	+	57900071	17	0	0	0	3	0.365095
CACACAGATCTACAAGGCCC (B0702-ex2g1)	chr2	-	57925212	13.5	2	2	0	2	0.158089
GATCTGAGCCGCGGTGTCCG (C0702-ex3g3)	chr2	-	42359128	13.5	2	2	0	2	0.142309
GATCTGAGCCGCGGTGTCCG (C0702-ex3g3)	chr2	-	44931692	11.25	3	3	0	2	0.172224
GATCTGAGCCGCGGTGTCCG (C0702-ex3g3)	chr2	-	45116717	11.25	3	3	0	2	0.144644
GATCTGAGCCGCGGTGTCCG (C0702-ex3g3)	chr2	-	54626146	13.5	2	1	1	3	0.098709
GATCTGAGCCGCGGTGTCCG (C0702-ex3g3)	chr2	+	55269338	13.5	2	2	0	2	0.166769

Supplementary Table 12.

Primers used in this study.

Primers	sequences
Primer **a** for large SV1 (chr3:41535938-61680677)	GGT GTG GTG TCT ATT AGC TCC
Primer **b** for large SV1 (chr3:41535938-61680677)	GGC AGG AAG GGA TAG GCT AAC
Primer **c** for large SV1 (chr3:41535938-61680677)	GAG ACT TGT TAC ATA AGG TGA GGA C
Primer **a** for large SV2 (chr15:44580927–44980927)	CTT CCA AGA TCT CTG CCC CTC
Primer **b** for large SV2 (chr15:44580927–44980927)	GCA GGG TTT CTC CAT TCT CTG G
Primer **c** for large SV2 (chr15:44580927–44980927)	GTG AAT GAG CCA TGG GCA CTG G
Primer **a** for large SV (chr3:39882164-39973392	ATA CCA AGT CAG TTT TCC TTC C
Primer **b** for large SV (chr3:39882164-39973392	ATC AGT CAG CCA GAA ATG ATG C
Primer **c** for large SV (chr3:39882164-39973392	TAA TTG TGA TGT TAG GGT GTC C
sgRNA for *B2M* knockout	CGCGAGCACAGCTAAGGCCA
sgRNA for *APP* knock in	AGAATTCCGACATGACTC
sgRNA for *B3GALT5* knockout	GTATATTTGCCTTCTGGTTC

Supplementary Fig. 1

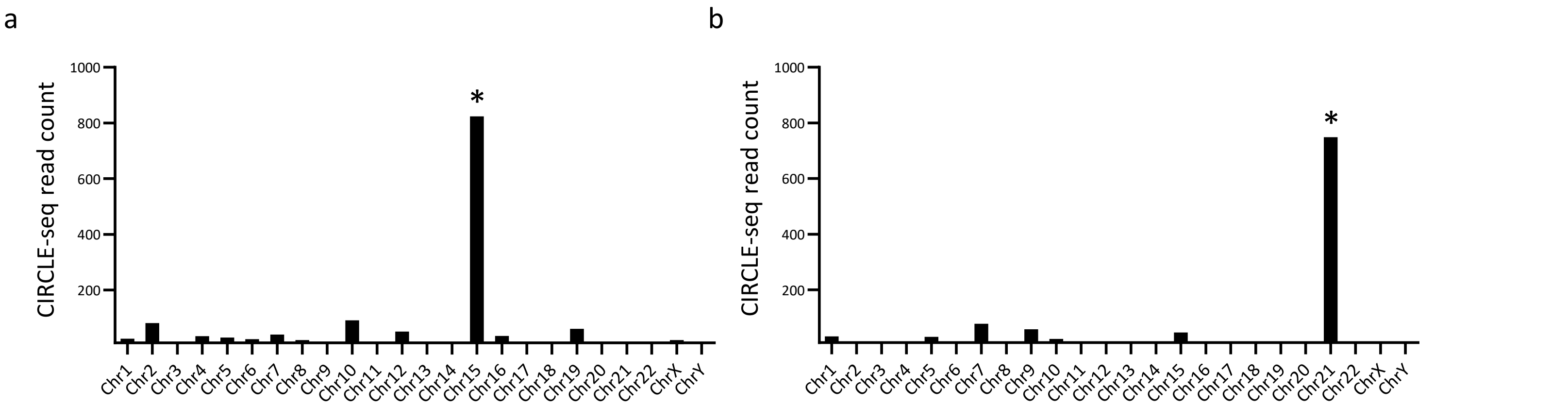

Supplementary Fig. 1. CIRCLE-seq detected off-target cleavage sites. (a) Histogram of CIRCLE-seq detected off-target sites of sgRNA (target to *B2M* gene) organized by chromosome, with bar heights representing CIRCLE-seq read counts. (b) Histogram of CIRCLE-seq detected off-target sites of sgRNA (target to *APP* gene). The asterisk * denotes the target site.

Supplementary Fig. 2

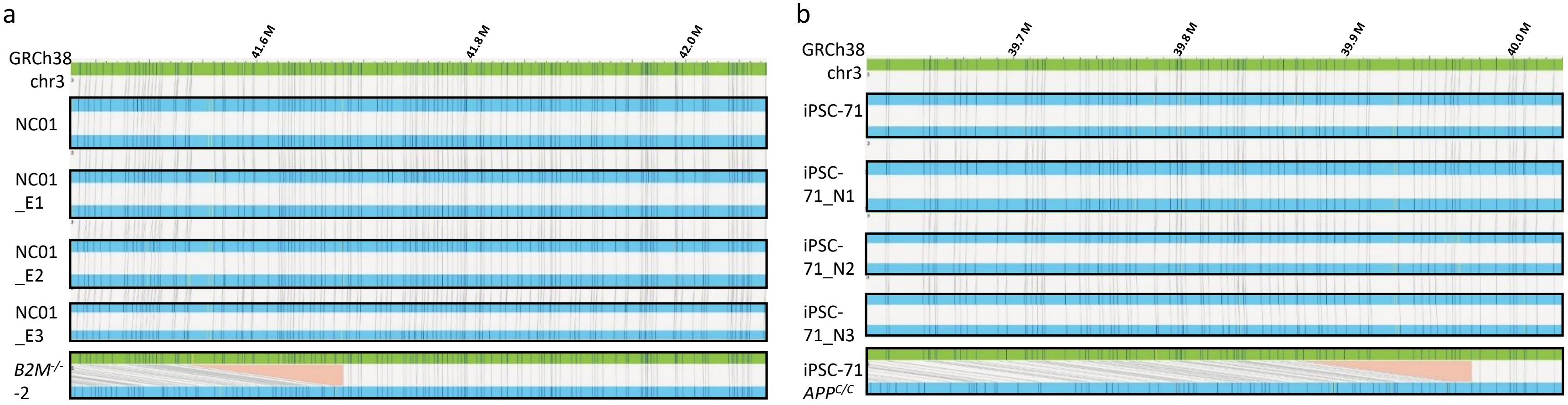

Supplementary Fig. 2. Detecting large structural variants that may be captured by optical genome mapping on a subject genome (blue) aligned to a reference genome (green). (a) Optical genome mapping data of parental (NC01), PiggyBac-mediated *ETV2i2* knock-in (NC01_E1, E2, and E3), and *B2M*$^{-/-}$-2 at the chr3: 41,427,045-42,075,423. (b) Optical genome mapping data of parental (iPSC-71), PiggyBac-mediated *NGN2* knock-in (iPSC-71_N1, N2, and N3), and *APP*$^{C/C}$ at the chr3: 39,611,790-40,030,052.

Supplementary Fig. 3

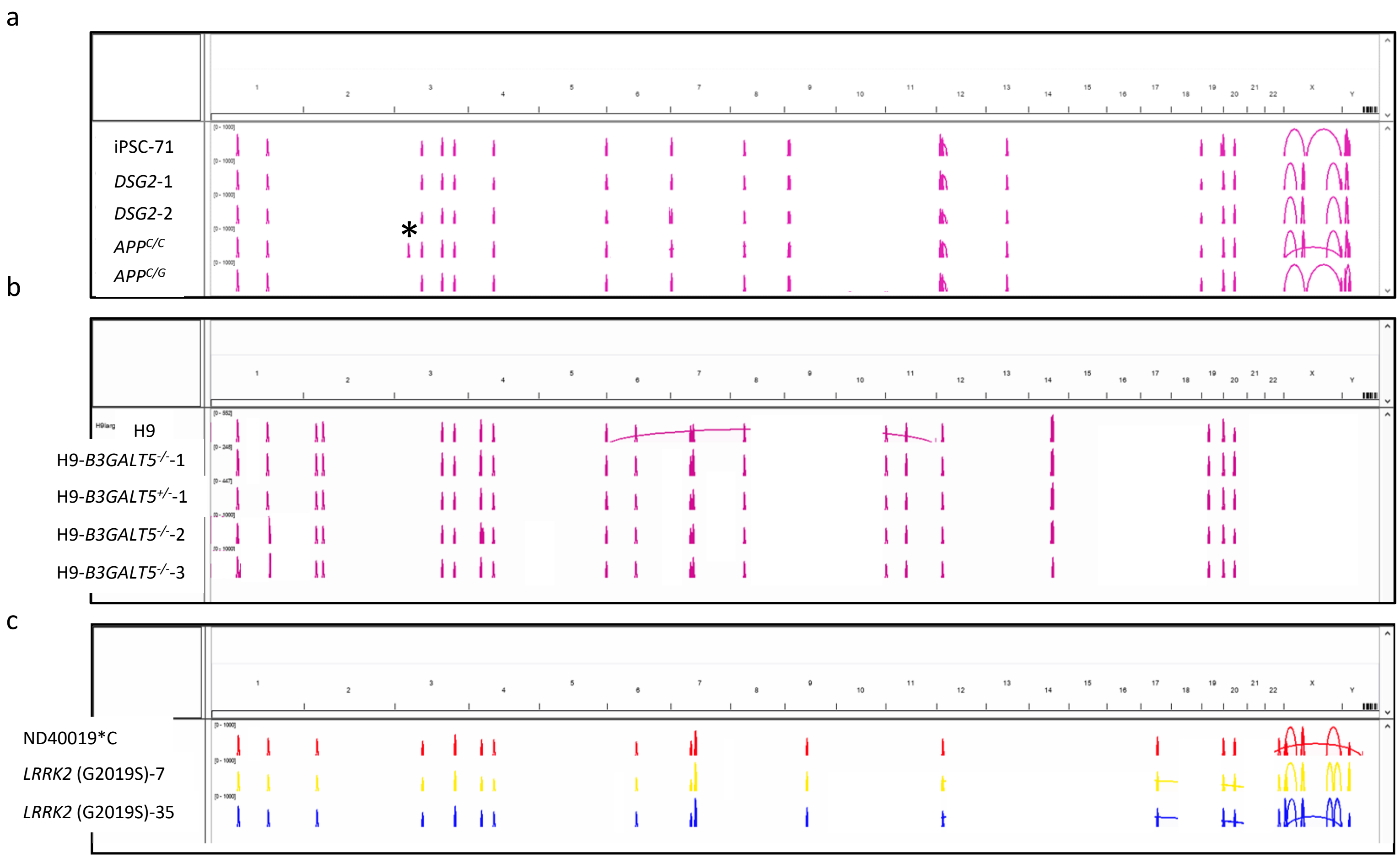

Supplementary Fig. 3. Large SV calls are constructed from linked-reads. (a) The large SV calls are constructed from linked reads of the parental iPSC-71 and the two single-cell clones of the *DSG2* (F531C) knock-in clones. (b) The large SV calls are constructed from linked-reads of the parental (H9) and the four single-cell clones of *B3GALT5* knockouts. (c) The large SV calls are constructed from linked-reads of the parental iPSC (ND40019*C) and the two single-cell clones of the *LRRK2* (G2019S) knock-in. Peaks represent the predicted large SV calls compared to GRCh38. There is no difference detected between parental and mutants. The asterisks * indicated large SVs on chromosomes 3 in *APP*$^{C/C}$.
