# Supplementary material for: Whole genomic analysis reveals atypical non-homologous off-target large structural variants induced by CRISPR-Cas9-mediated genome editing
Source: Nat Commun. 2023 Aug 25;14:5183. doi: 10.1038/s41467-023-40901-x (PMC10457329; doi:10.1038/s41467-023-40901-x)
Supplement: Supplementary file 2 — Reporting Summary [file 41467_2023_40901_MOESM2_ESM.pdf]

nature portfolio

Corresponding author(s): John Yu

Last updated by author(s): Aug 15, 2023

Reporting Summary

Nature Portfolio wishes to improve the reproducibility of the work that we publish. This form provides structure for consistency and transparency in reporting. For further information on Nature Portfolio policies, see our Editorial Policies and the Editorial Policy Checklist.

Statistics

For all statistical analyses, confirm that the following items are present in the figure legend, table legend, main text, or Methods section.

n/a	Confirmed	
x		The exact sample size (*n*) for each experimental group/condition, given as a discrete number and unit of measurement
x		A statement on whether measurements were taken from distinct samples or whether the same sample was measured repeatedly
x		The statistical test(s) used AND whether they are one- or two-sided<br>*Only common tests should be described solely by name; describe more complex techniques in the Methods section.*
x		A description of all covariates tested
x		A description of any assumptions or corrections, such as tests of normality and adjustment for multiple comparisons
	x	A full description of the statistical parameters including central tendency (e.g. means) or other basic estimates (e.g. regression coefficient) AND variation (e.g. standard deviation) or associated estimates of uncertainty (e.g. confidence intervals)
x		For null hypothesis testing, the test statistic (e.g. *F*, *t*, *r*) with confidence intervals, effect sizes, degrees of freedom and *P* value noted<br>*Give P values as exact values whenever suitable.*
x		For Bayesian analysis, information on the choice of priors and Markov chain Monte Carlo settings
x		For hierarchical and complex designs, identification of the appropriate level for tests and full reporting of outcomes
x		Estimates of effect sizes (e.g. Cohen's *d*, Pearson's *r*), indicating how they were calculated

Our web collection on statistics for biologists contains articles on many of the points above.

Software and code

Policy information about availability of computer code

Data collection	Cas-OFFinder (http://www.rgenome.net/cas-offinder/), CRISTA (https://crista.tau.ac.il/), Elevation (https://crispr.ml/)
Data analysis	Long Ranger (v2.2.2), Loupe (v2.1.1), Bionano Slove (v3.3, v3.6.1 and v3.7.01)

For manuscripts utilizing custom algorithms or software that are central to the research but not yet described in published literature, software must be made available to editors and reviewers. We strongly encourage code deposition in a community repository (e.g. GitHub). See the Nature Portfolio guidelines for submitting code & software for further information.

Data

Policy information about availability of data

All manuscripts must include a data availability statement. This statement should provide the following information, where applicable:

- Accession codes, unique identifiers, or web links for publicly available datasets
- A description of any restrictions on data availability
- For clinical datasets or third party data, please ensure that the statement adheres to our policy

The 10x Genomics sequencing data of genomes generated in this study have been deposited in the NCBI SRA database under accession code PRJNA943092 (https://www.ncbi.nlm.nih.gov/sra/PRJNA943092). All other relevant data are available from the corresponding author upon request. Source data are provided with this paper.

Research involving human participants, their data, or biological material

Policy information about studies with human participants or human data. See also policy information about sex, gender (identity/presentation), and sexual orientation and race, ethnicity and racism.

Reporting on sex and gender	No human participants were recruited in this study.
Reporting on race, ethnicity, or other socially relevant groupings	No human participants were included in this study.
Population characteristics	No human participants were recruited in this study.
Recruitment	No human participants were included in this study.
Ethics oversight	No human participants were included in this study.

Note that full information on the approval of the study protocol must also be provided in the manuscript.

Field-specific reporting

Please select the one below that is the best fit for your research. If you are not sure, read the appropriate sections before making your selection.

[x] Life sciences [ ] Behavioural & social sciences [ ] Ecological, evolutionary & environmental sciences

For a reference copy of the document with all sections, see nature.com/documents/nr-reporting-summary-flat.pdf

Life sciences study design

All studies must disclose on these points even when the disclosure is negative.

Sample size	No statistical analysis was performed to predetermine sample size. The sample sizes used in this study were determined based on the availability of sample.
Data exclusions	No data were excluded from the analyses.
Replication	All of the experiments in this manuscript were replicated and for biochemical and relevant analysis were repeated in biological triplicates as described in the figure legends.
Randomization	The samples were not randomized in our study. This study was to confirm the genome integrity by whole genome sequencing, therefore it was impossible to rearrange samples randomly.
Blinding	Blinding was not used in our study. This study was to identify and characterize genome integrity by whole genome sequencing and cell-based approaches. The performance bias (blinding of participants) do not occur in our studies. But, the technical personnel who performed 10X Linked-read sequencing and optical genome mapping did not know the identity of samples, but the code.

Reporting for specific materials, systems and methods

We require information from authors about some types of materials, experimental systems and methods used in many studies. Here, indicate whether each material, system or method listed is relevant to your study. If you are not sure if a list item applies to your research, read the appropriate section before selecting a response.

Materials & experimental systems

n/a	Involved in the study	
[ ]	[x]	Antibodies
[ ]	[x]	Eukaryotic cell lines
[x]	[ ]	Palaeontology and archaeology
[x]	[ ]	Animals and other organisms
[x]	[ ]	Clinical data
[x]	[ ]	Dual use research of concern
[x]	[ ]	Plants

Methods

n/a	Involved in the study	
[x]	[ ]	ChIP-seq
[ ]	[x]	Flow cytometry
[x]	[ ]	MRI-based neuroimaging

Antibodies

Antibodies used	The antibodies used in this study:<br>HLA-ABC Monoclonal Antibody (W6/32), FITC, eBioscience, 11-9983-42, Lot 2132555, 1:200

	Mouse IgG2a kappa Isotype Control (eBM2a), FITC, eBioscience™, 11-4724-42, 1:200
Validation	HLA-ABC: https://www.thermofisher.com/antibody/product/HLA-ABC-Antibody-clone-W6-32-Monoclonal/11-9983-42<br>Mouse IgG2a kappa Isotype Control: https://www.thermofisher.com/antibody/product/Mouse-IgG2a-kappa-clone-eBM2a-Isotype-Control/11-4724-42

Eukaryotic cell lines

Policy information about cell lines and Sex and Gender in Research

Cell line source(s)	H9 (NSC-H9, WiCell), NC01 (generateded in our lab), iPSC-71 (material transfer from Joseph C. Wu, Stanford Cardiovascular Institute), ND40019*C (doi:10.1186/s13287-021-02585-2)
Authentication	Cell morphology, Immunofluorescence staining (SSEA4, Oct4, TRA-1-60, TRA-1-81)
Mycoplasma contamination	Cells used in this study are routinely screened using PCR. All tests for mycoplasma in the cells used in this study were negative.
Commonly misidentified lines<br>(See ICLAC register)	No commonly misidentified cell line used in this study.

Flow Cytometry

Plots

Confirm that:

- [x] The axis labels state the marker and fluorochrome used (e.g. CD4-FITC).
- [x] The axis scales are clearly visible. Include numbers along axes only for bottom left plot of group (a 'group' is an analysis of identical markers).
- [x] All plots are contour plots with outliers or pseudocolor plots.
- [x] A numerical value for number of cells or percentage (with statistics) is provided.

Methodology

Sample preparation	Culture cells were dissociated with Accutase and prepared for FACS staining in PBS-2% FBS buffer
Instrument	Data were acquired with SONY SA3800 Spectral Cell analyzer.
Software	The data were analyzed with FlowJo 10.8.1.
Cell population abundance	We did not perform flow cytometry sorting in this study.
Gating strategy	The gating strategy are described in detailed in the Source Data file. The single cell gating strategy was used to exclude cell aggregates.

- [x] Tick this box to confirm that a figure exemplifying the gating strategy is provided in the Supplementary Information.
